# Increased expression of PD-1 and PD-L1 in oral lesions progressing to oral squamous cell carcinoma: a pilot study

**DOI:** 10.1038/s41598-020-66257-6

**Published:** 2020-06-16

**Authors:** Kanan Dave, Aiman Ali, Marco Magalhaes

**Affiliations:** 1 Oral Pathology and Oral Medicine, Faculty of Dentistry, University of Toronto, Toronto Ontario, Canada; 2 Cancer Invasion and Metastasis Laboratory, Faculty of Dentistry, University of Toronto, Toronto Ontario, Canada; 3 Sunnybrook Health Sciences Centre, Department of Dental and Maxillofacial Sciences, Toronto Ontario, Canada

**Keywords:** Prognostic markers, Cancer, Oral conditions, Oral medicine, Oral pathology

## Abstract

Oral cancer is a devastating disease and is commonly preceded by a range of oral premalignant disorders. We investigated the expression of PD-1 and PD-L1 in oral epithelial dysplasia (OED) that progressed to oral squamous cell carcinoma (OSCC) compared to non-progressing dysplasia. 49 oral biopsies were analyzed, including 19 progressing cases, 20 cases did not progress, and 10 OSCC. Samples were stained with monoclonal antibodies for PD-1 and PD-L1, followed by conventional peroxidase reaction immunohistochemistry (IHC) imaged under light microscopy or fluorescent immunohistochemistry (FIHC) imaged using a confocal microscope. Images were analyzed using a novel semi-automated analysis protocol. PD-1/PD-L1 expression was assessed at the epithelium/tumor cells (TC) and at inflammatory cells in lamina propria. Our results show a significant increase in PD-L1 expression in progressing compared to non-progressing dysplasia. Using FIHC, we showed increased PD-L1 expression, increased nuclear density in progressing dysplasia and a better interobserver agreement compared with IHC. We developed a new FIHC-based quantitative method to study PD-1/PD-L1 expression in FFPE samples and showed that PD-L1 is highly expressed in premalignant lesions progressing to cancer. Our results suggest that immunomodulation via PD-L1/PD-1 pathway occurs prior to malignant transformation.

## Introduction

Oral squamous cell carcinoma (OSCC) is a multifactorial malignant disease arising from oral mucosa and carries a poor prognosis that has changed minimally in the past several decades^1^. In addition to poor survival rates, and treatment may result in high morbidity since the disease affects facial tissues, significant esthetic, and functional loss after treatment. OSCC is commonly preceded by a range of tissue and cellular alterations in the form of oral epithelial dysplasia (OED) and are classified under the umbrella of Oral Potentially Malignant Disorders (OPMD) of the oral mucosa^2^. OED represents a heterogeneous group of conditions that are graded from mild to severe depending on the extent of abnormalities in the tissue^3,4^ and carries an overall risk of malignant transformation of up to 36%^5,6^. A 10-year review of the Toronto Oral Pathology Service (TOPS) showed that OED are more prevalent than benign and malignant tumors of the oral cavity combined^7^. Considering the high incidence of OED, malignant transformation represents a significant health problem with thousands of cases of OSCC diagnosed yearly. Consequently, predicting transformation in premalignant lesions would facilitate earlier cancer  treatment and could significantly decrease morbidity and mortality^8,9^.

OSCC is commonly associated with a dense inflammatory infiltrate, and our laboratory has previously shown that OSCC patients show a marked increase in pro-inflammatory cytokines^10^ that can promote invasion of OSCC cells *in vitro*^10,11^. In the context of cancer-associated inflammation, the immune checkpoint system has been increasingly studied and is frequently activated in cancer to suppress antitumor immune responses^12,13^. Programmed cell death protein-1 (PD-1) is a member of extended CTLA-4 (cytotoxic T lymphocyte-associated protein 4) family of T regulators^14^ and is primarily expressed at the membrane of T lymphocytes. PD-1 ligands (PD-L1/PD-L2) are cell surface ligands found on antigen-presenting cells and epithelial cells. Interaction of PD-1 with its ligands induces anergy of T cells, effectively inhibiting T cell activation, proliferation, and production of cytokines^14–16^, which is essential for immune homeostasis and tolerance in healthy tissue. PD-L1 is highly expressed in different tumors, including melanomas, lymphomas, and renal cell carcinoma^17–19^ and the presence of PD-L1 + cells in these tumors correlates with poor prognosis^18,19^. A recent systematic review concluded that anti-PD-1 medications (Sitravatinib and Nivolumab) or PD-L1 (Pembrolizumab) for advanced head and neck cancer have shown promising results with increased survival in patients with recurrent/metastatic HNSCC compared to standard chemotherapeutic treatment^20^. PD-1/PD-L1 is overexpressed in OSCC^21,22^ but little is known about the role of this pathway in oral dysplasia. Maruse *et al*. has shown that PD-1/PD-L1 expression is associated with nodal metastasis and poor prognosis in OSCC^23^. A recent retrospective study showed that increased CD163 and PD-L1 expression at the lamina propria are associated with an increased risk of malignant transformation in oral dysplasia, but only 8 cases of transformation were analyzed in the study^24^. Among the challenges of interpreting the expression of PD-1 and PD-L1 are the inconsistencies in staining and quantification^25–27^. We hypothesized that a new fluorescent-based analysis of PD-1 and PD-L1 expression could improve quantification and interobserver agreement compared to conventional IHC quantification. Further, we hypothesized that this new technique could be used to show increased PD-1/PD-L1 expression in progressing oral lesions. To test these hypotheses, we have evaluated 19 cases of progressing lesions and 20 cases of control (non-progressing) and compared PD-1 and PD-L1 expression using conventional immunohistochemistry (IHC) and fluorescent immunohistochemistry (FIHC) technique based on a semi-automated analysis algorithm. Both IHC and FIHC techniques showed increased expression of PD-L1 in oral lesions that progressed to cancer, suggesting that PD-1/PD-L1 expression precedes malignant transformation. FIHC analysis resulted in better stratification of differences according to diagnosis using mean fluorescence intensity (MFI) and overall expression (MFI x area) and improved interobserver agreement. Our new quantification of FIHC has the potential to improve clinical assessment of PD-1/PD-L1 expression to define clinical treatment.

## Materials and methods

### Research ethics

All methods and experiments are following the University of Toronto research guidelines. Ethical approval was obtained from the University of Toronto research ethics board #36029. The research ethics committee of the University of Toronto waived the informed consent.

### Sample selection

All cases were selected from the archives of the Toronto Oral Pathology Service (TOPS) at the Faculty of Dentistry, University of Toronto, using a custom made FileMaker database. We reviewed cases received between January of 2008 and December of 2017, including 880 cases with a diagnosis of OSCC and 75 cases with a previous biopsy showing dysplasia in the same area. For the progressing group, we reviewed these 75 cases and selected 20 formalin Fixed Paraffin-Embedded (FFPE) samples that subsequently developed into OSCC following the criteria: 1- minimum of 5 years of follow up available, 2- enough material for analysis (progressing cases), 3- no significant artefacts in the sections. After analysis, 1 case was removed due to the presence of significant artefacts on processing even after multiple deeper sections cut. Therefore the total number of progressing cases was 19. For the control group (**Non-progressing group)**, we randomly selected 20 cases of dysplasia (mild, moderate, severe) that did not show evidence of progression to OSCC (non-progressing) after 7 years based on our database analysis matching patient/lesion characteristics (age, gender, location) and diagnosis. 10 cases of OSCC were also included, 5 of which were from patients in the progressing dysplasia group. All the slides were reviewed by MM and AA and diagnoses was divided into a two-tier system for dysplasia (**Low-grade - LGD and High-Grade dysplasias - HGD**), hyperkeratosis without dysplasia and OSCC. The clinical notes and histopathological features of the cases of hyperkeratosis were reviewed and do not fit the criteria for the diagnosis of Proliferative Verrucous Leukoplakia.

### Immunohistochemistry (IHC)

Four-micrometer sections were prepared from each paraffin block for subsequent immunohistochemistry with PD-1 and PD-L1 using Ventana Medical Systems (Arizona-USA). Staining for anti-PD-L1, anti-PD-1 clones were performed on the Ventana Benchmark Ultra automated staining platform according to the manufacturer’s protocol.

### IHC analysis

**Figure 1:**
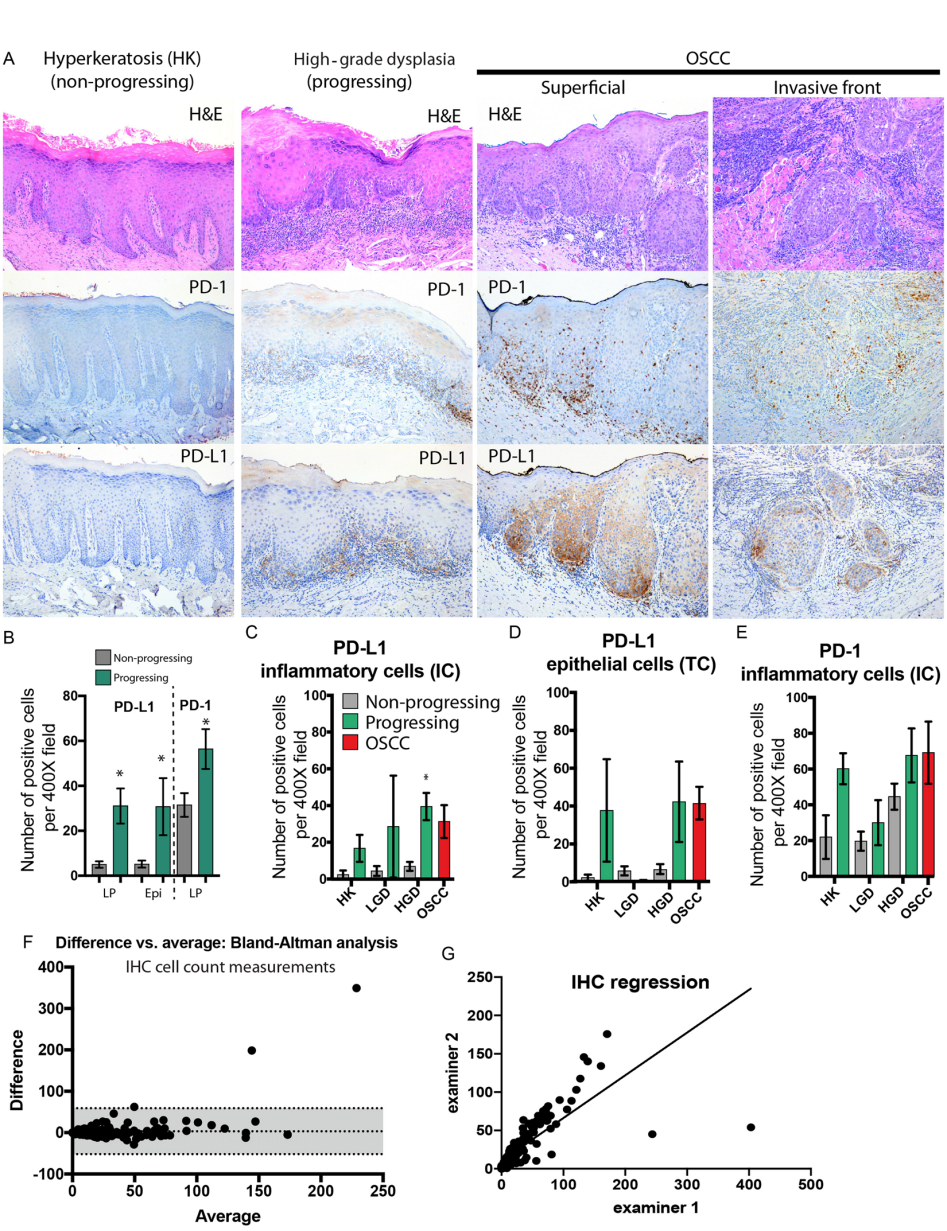
Expression of PD-1 and PD-L1 based on IHC cell count analysis. (**A**) Representative microscopic images of samples stained for PD-1 and PD-L1 using a commercially available Ventana system and imaged using conventional light microscopy. Left panels represent hyperkeratosis (non-progressing), middle panels are progressing severe dysplasia and right panels are from a squamous cell carcinoma. The number of positive cells in each sample was calculated after the analysis of 5 high power fields (400×) by 2 independent examiners. **(B)** Number of PD-1+ IC and PD-L1 IC/TC cells per high power field in progressing (green bars) versus control (grey bars) (n = 49, *P < 0.05 between progressing and control) **(C)** Number of PD-L1 + IC per high power field (**D)** Number of PD-L1 + IC per high power field (n = 49, *P < 0.05 between progressing and control). **(E)** Number of PD-1+ IC per high power field. **(F)** Bland-Altman analysis, difference versus average, between two examiners (bias of 3.256 ± 28.58 and a 95% limit of the agreement between −52.8 and +59.3). **(G)** Linear regression of the scores of the two examiners (slope of 0.5574 ± 0.03144 and R^2^ = 0.5904).

PD-1 and PD-L1 IHC slides were de-identified and analyzed by two blinded examiners (KD, AA). The results were compiled and analyzed by M.M. Slides were evaluated under 40X magnification and five different fields from the representative areas were selected for all cases. Any level of membranous/cytoplasmic cell positivity in each field was recorded. PD-1 and PD-L1 positive cells were counted in subepithelial areas up to 60 µm from the basement membrane, corresponding to the superficial lamina propria of oral mucosa. Epithelial expression of PD-L1 was evaluated in the basal and spinous layer of all cases **(**Fig. 1A**)**.

### Fluorescent immunohistochemistry (FIHC)

Five-micron sections were prepared from each FFPE block. Slides were heated for 30 minutes up to 60 °C and then immersed in antigen retrieval buffer (100X Citrate Buffer pH 6.0, Abcam-CA) at 98 °C for one hour. Slides were washed with warm water for one minute after cooling down in room temperature, dried, marked with PapPen and covered with TBS-T. Then Triton (X100-Bioshop. 75 µl diluted in 15 ml PBS) was added to each slide for 5 minutes. Samples were washed with TBS-T three times 5 minutes each and then blocking buffer (Sea Block Serum free-PBS abcam-UK) was added for 2 hours. Primary antibodies anti-PD-1 (clone NAT 105 clone, Mouse ab52587) and anti-PD-L1 (clone 28-8, Rabbit Abcam ab205921) were added at 1:100, and 0.15:100 dilution respectively. Samples were incubated overnight at 4 °C and washed next day with TBS-T three times 5 minutes each and then secondary antibodies (Alexa Fluor 488-mouse ab150105, and 568-Rabbit ab175694 from Abcam) were incubated at room temperature for one hour. Slides were washed with TBS-T three times, slides were kept 5 minutes in TBS-T each wash. After third wash, DAPI (Thermo Fisher Scientific, USA) was added for 30 minutes followed by washing. Slides were mounted and covered using ProLong Diamond Antifade Mountant (Invitrogen) mounting media. All samples were imaged using confocal microscopy the same day and then analyzed by Volocity 3D Image Analysis Software (PerkinElmer, USA).

### FIHC Data analysis

**Figure 2:**
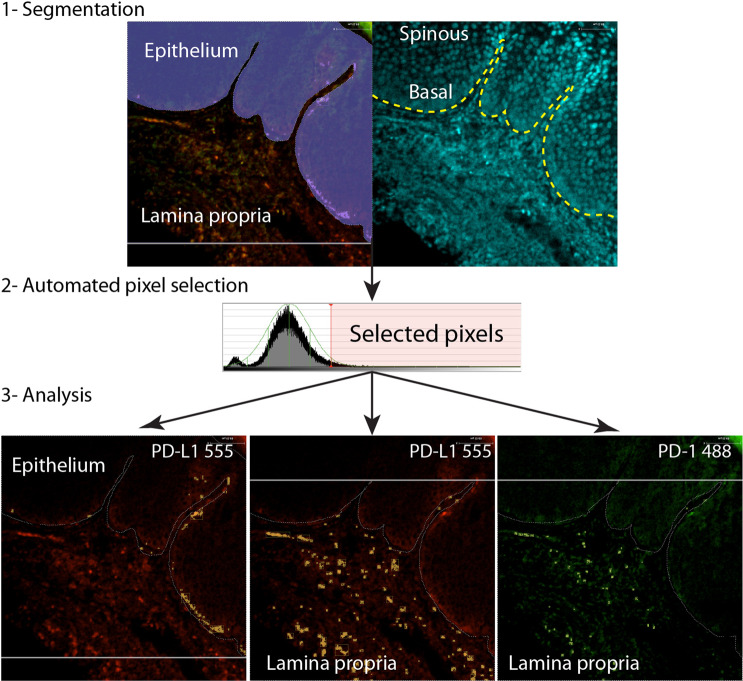
FIHC image segmentation and quantification. High-resolution confocal images were manually segmented using DAPI channel (1). The positive cells were identified using the standard deviation of the pixel intensity. Pixels above three standard deviations of the MFI were selected (2) and artefacts excluded by size and shape. The area of the positive cells and MFI was calculated for each specific segment: lamina propria, basal cell layer and spinous layer (3). The expression was calculated as the product of Area X MFI.

Ten images of each slide were taken at 10x objective and 1.6X magnification lenses using a Quorum Spinning Disk Confocal microscope (Quorum Technologies Inc., Canada) and the exact imaging settings. The morphological features were identified using DAPI. The images were segmented manually to create regions of interest (ROI) around the inflammatory infiltrates in lamina propria, basal epithelial layer and spinous layer. OSCC lesions showed islands and cords of epithelial cells invading the adjacent connective tissue of which we segmented tumor versus stroma only (Fig. 2). For all specimens, the walls and the lumen of large capillaries, small and medium-sized blood vessels within the lamina propria or stroma were removed from the ROI. PD-1 and PD-L1 positive cells were identified using an automated protocol based on pixel intensity. Briefly, pixels above three standard deviations (SD) of the mean intensity of the channels were selected. The mean fluorescence intensity (MFI) and area of the positive pixels were calculated for each image and the average of at least 5 images was considered for each specimen. The images were analyzed by 2 blinded examiners (AA, KD) using the same image protocol and the results were reviewed and analyzed by MM. There were images with significant artefactual changes and background autofluorescence, and these were removed from the analysis. Nuclear quantification was performed using Definiens Tissue Studio 4.0, using the DAPI channel and the standard nuclei detection tool.

### Statistical analysis

The results were analyzed using one-way ANOVA (expression according to diagnosis) or two-way ANOVA (progression and diagnosis) followed by Fisher’s LSD for multiple comparisons. Datasets with 2 groups were analyzed using t-tests. Bland-Altman plots and linear regression were used to compare the distribution of the scores between examiners in IHC and FIHC samples. Matched premalignant and malignant cases were analyzed using paired t-tests. Patient characteristics were analyzed using independent samples Kruskal-Wallis test or Mann-Whitney U test. Statistical significance was determined as P < 0.05. All statistical analyses were performed using GraphPad Prism 7.0 and IBM SPSS version 25 and all figures were prepared using Adobe Illustrator CC 2019.

## Results

### Patient characteristics

The demographics of the groups and characteristics of the patient cases can be seen in Table 1. The mean age was 63.75 years with male:female ratio of 1.2:1. There were no significant differences in the age distribution between diagnoses (P < 0.487) but the average age of the progressing group (67 ± 10) was higher than control group (58 ± 6)(P < 0.04). The age differences were mostly restricted to the progressing HK group that had an average of 77± 6 years compared to 57 ± 8 of the control HK group. The most common involved site was tongue (n = 26, 53% of cases) followed by buccal mucosa (n = 8, 16.3%). The average time of progression to OSCC was ~3.05 years, confirmed with follow up biopsies showing cancer in the same area.

**Table 1:** Characteristics of the patient cases included in the study.

		Hyperkeratosis		Low-grade dysplasia		High-grade dysplasia		OSCC
		Control	Progressing	Control	Progressing	Control	Progressing	
Age (Mean)		57+/− 8	77+/− 8	58+/− 8	63+/− 10	58+/− 8	63+/− 8	66+/− 14
Sex	Male	3 (60%)	3 (60%)	5 (71.4%)	1 (16.7%)	3 (37.5%)	5 (62.5%)	7 (70%)
	Female	2 (40%)	2 (40%)	2 (28.6%)	5 (83.3%)	5 (62.5%)	3 (37.5%)	3 (30%)
Location	Tongue	1 (2%)	3 (6%)	3 (6.1%)	4 (8.2%)	5 (10.2%)	4 (8.2%)	6(12.2%)
	FOM	0	0	1 (2%)	1 (2%)	0	0	0
	Palate	1 (2%)	1 (2%)	2 (4.1%)	0	1 (2%)	1 (2%)	0
	Buccal mucosa and lip	1 (2%)	0	1 (2%)	1 (2%)	1 (2%)	3 (6.1%)	3 (6.1%)
	Gingiva and Retromolar pad	2 (4.1%)	1 (2%)	0	0	1 (2%)	0	1 (2%)
Total	49	5	5	7	6	8	8	10

### PD-1 and PD-L1 expression in dysplasia and OSCC using IHC

The current protocol for assessing PD-1/PD-L1 is based on calculation of percentage or numbers of positive tumor/dysplastic cells (TC) and inflammatory cells (IC). We have calculated the number of positive TC and IC in progressing and non-progressing lesions as well as OSCC. Our results show positive TC/IC PDL-1+ and IC PD-1+ cells in all specimens analyzed and progressing high-grade dysplasia (HGD) and OSCC with the highest number of positive PD-1/PD-L1 cells while non-progressing HK had the lowest expression (Fig. 1A). There was significant heterogeneity of PD-1 and PD-L1 staining between fields within the samples. We observed a significant increase in TC/IC PDL-1+ and IC PD-1+ in cases that progressed to carcinoma (PD-L1 TC/IC P < 0.01, PD-1 IC P < 0.02) (Fig. 1B). Evaluation of positive cells according to diagnosis revealed a significant increase in PD-L1 IC expression in progressing HGD **(**Fig. 1C**)** and a trend towards increased expression in progressing HK and LGD, albeit not statistically significant (P < 0.38, P < 0.12). There was also a trend towards increased expression of PD-L1 TC in HK (P < 0.17) and HGD (P < 0.06) **(**Fig. 1D**)** and there were no significant differences observed in PD-1 expression (HK P < 0.06, LGD P < 0.58, HGD P < 0.11) **(**Fig. 1E**)**. We observed a large standard error of the mean in several groups (e.g. PD-L1 LGD, PD-L1 HK and HGD) and we performed a Bland-Altman analysis **(**Fig. 1F**)** to determine if the error could be explained by interobserver differences. The results showed a bias of 3.256 ± 28.58 and a 95% limit of the agreement between −52.8 and +59.3 with marked differences between the observers in higher scoring samples. Linear regression analysis revealed a slope of 0.5574 ± 0.03144 and R^2^ = 0.5904 (Fig. 1G**)**.

Based on these results, we hypothesized that a more reproducible quantification system could improve the differentiation of PD-1 and PD-L1 expression in these samples. We have developed a new FIHC-based method and the results are described next.

### PD-1/PD-L1 expression using FIHC

**Figure 3:**
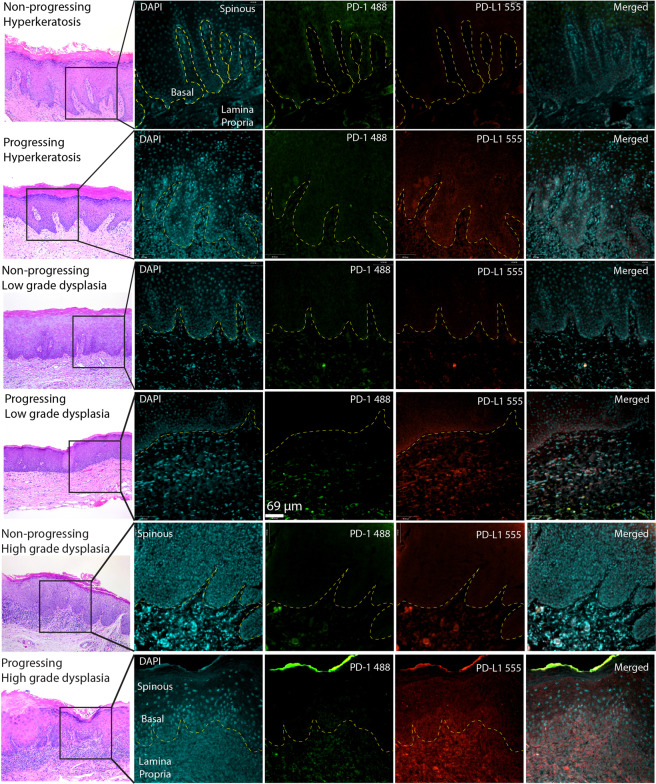
Representative images of non-progressing and progressing lesions of hyperkeratosis, mild and severe dysplasia. The samples were stained using conventional H&E and FIHC and imaged using a spinning disk confocal microscope (see Materials and methods). The right panels show the expression of PD-1 and PD-L1 and the segmented areas used to calculate expression as Area × MFI. Images are representative of 10 images from each sample and a total 10 HK, 9 LGD, 10 HGD and 10 samples of OSCC.

**Figure 4:**
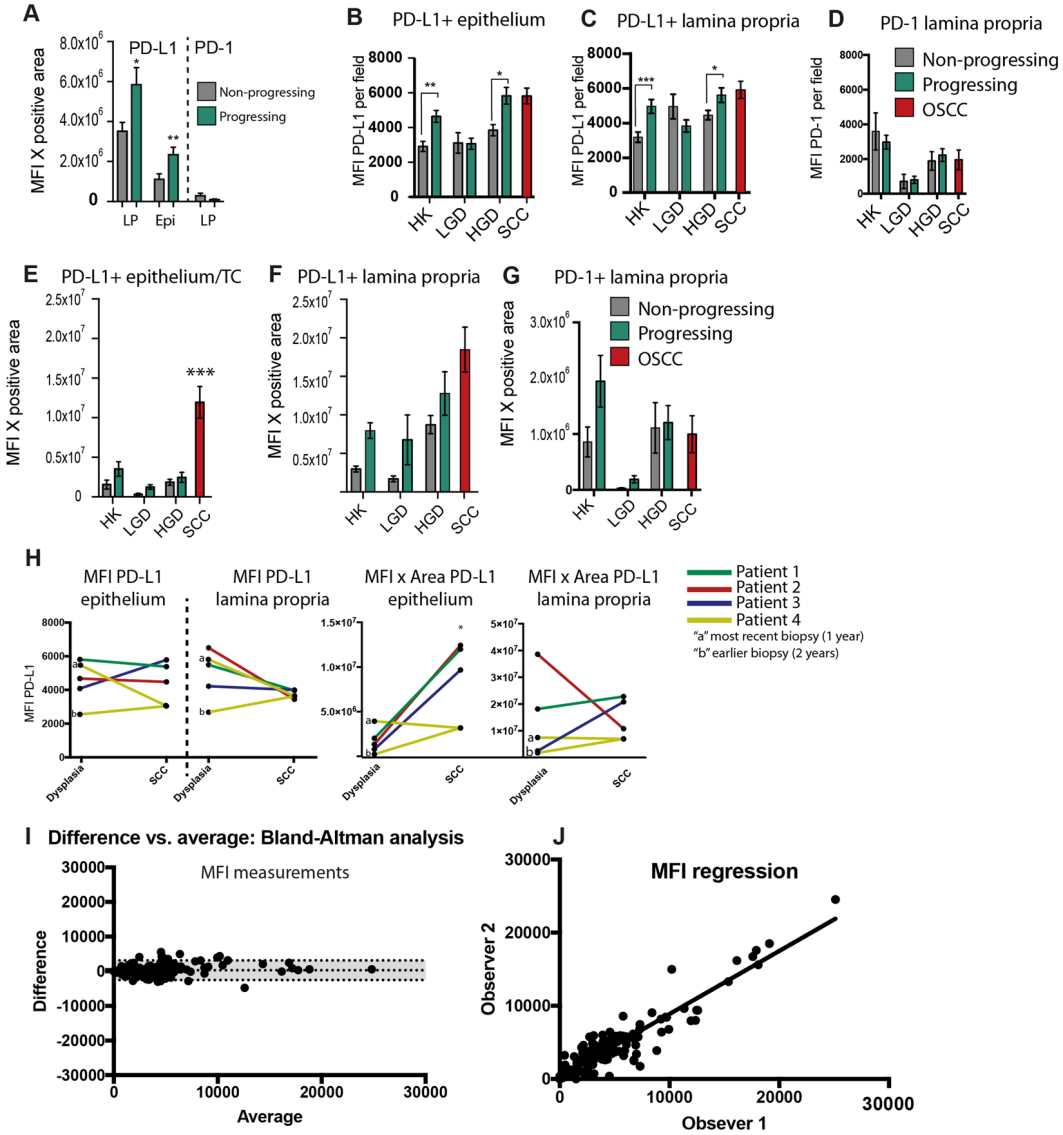
Quantification of PD-1 and PD-L1 in FIHC images. (A) Expression of PD-1+ at the lamina propria (LP) and PD-L1 + (LP and epithelium) calculated as MFI × area in progressing (green bars) versus control (grey bars) (*P < 0.05, ** P < 0.01). MFI of PD-L1 was calculated at the epithelium **(B)** and lamina propria **(C)** and PD-1 at the lamina propria **(D)**. Expression of PD-L1 was calculated as the product of MFI and average positive area at the epithelium **(E)** and lamina propria **(F)** and PD-1 at the lamina propria **(G)**. A total of 5 images per sample were analyzed, and the average of the results is shown in the bar graph - progressing (green bars) versus control (grey bars)(*P < 0.05, ** P < 0.01). **(H)** Differences in expression of PD-L1 (MFI × Area) between matched premalignant and OSCC patients. One case has 2 previous biopsies and “a” represents the most recent biopsy (1 year) and “b” represents the earlier biopsy (2 year). There are no significant differences between the groups (paired t-tests between OSCC and premalignant cases, P > 0.4) **(I)** Bland-Altman analysis, difference versus average, between two examiners (bias of 289 ± 1446 and a 95% limit of the agreement between −2546 and +3124). **(J)** Linear regression of the scores of the two examiners (slope of 0.8615 ± 0.02436 and R^2^ = 0.8742).

There are important limitations to operator-based IHC quantification of protein expression, including non-linear distribution of protein staining intensity and decreased interobserver agreement^25^. To address these shortcomings, we created a novel FIHC-based protocol to quantify PD-1 and PD-L1 in histopathological samples. The images were acquired using a confocal microscope and analyzed using a semi-automated protocol that selected positive pixels based on the standard deviation of the pixel intensity as described in (Fig. 2**)**. We applied this novel protocol to the same cases evaluated by IHC (Fig. 1**)** and our results show a similar distribution of PD-L1 + cells compared to IHC but reduced detectable expression of PD-1. PD-L1 expression was seen primarily at the basal epithelial layer, inflammatory cells at the lamina propria, tumor-associated inflammatory cells and occasional intraepithelial inflammatory cells **(**Fig. 3**)**. PD-L1 was not expressed in the spinous layer of the epithelium **(**Fig. 3**)**. There was very limited expression of PD-1, restricted to scattered inflammatory cells at the lamina propria. There was an increase in PD-L1 expression in progressing lesions (lamina propria, P < 0.0148, Epithelium P < 0.0072) but no significant changes in expression of PD-1 (P < 0.1312) **(**Figs. 3 and 4A**)**.

### FIHC-based quantification of expression

We have analyzed the expression PD-1 and PD-L1 based on the mean fluorescent intensity (MFI) (Fig. 4A–D) as well as the overall expression of the protein quantified as the product of MFI and the average positive area to compensate for large variations in positive area between the groups **(**Fig. 4E–G**)**. Progressing lesions of HK and HGD showed a consistently higher PD-L1 MFI at basal epithelial cells (P < 0.02 and P < 0.0006) and inflammatory cells (P < 0.006 and P < 0.02) **(**Fig. 4B,C**)**. There were no differences in PD-1 MFI between progressing and control cases **(**Fig. 4D**)**. OSCC showed the highest expression of PD-L1 (P < 0.0001 between SCC and all the other groups) and this is mostly due to the larger average positive areas in OSCC cases **(**Fig. 4E). There was a trend towards increased PD-L1 expression in progressing HK, LGD and HGD (ANOVA P < 0.001, LSD test: HK P < 0.11, LGD P < 0.11, HGD P < 0.13) **(**Fig. 4F**)** but no significant differences in the expression of PD-1 **(**Fig. 4G**)**. We have analyzed the expression of PD-1 and PD-L1 in premalignant and matching OSCC cases. There were 5 samples from 4 patients analyzed (1 patient had 2 previous biopsies). The MFI results show comparable PD-L1 expression (PD-L1 epithelium P < 0.80, PD-L1 LP P < 0.16) **(**Fig. 4H**)** and the Area X MFI analysis show a significant increase in PD-L1 expression at OSCC cells compared to premalignant lesions in 4 out of 5 cases (PD-L1 epithelium P < 0.04, PD-L1 LP P < 0.80).

There are no statistical differences between the scores of examiners 1 and 2 (examiner 1 = 4009 ± 302, examiner 2 = 3680 ± 276.7, P < 0.4). A Bland-Altman analysis **(**Fig. 4I**)** was performed to analyze the distribution of the scores of the two observers. The results showed a bias of 289 ± 1446 and a 95% limit of the agreement between −2546 and +3124. The 95% limit of the agreement is relatively narrower compared to the IHC analysis that resulted in 95% limit of the agreement (−52.76, +59.27) while the averages of the examiner were 30.98 ± 3.004 and 27.73 ± 2.179 (Fig. 1). Linear regression analysis revealed a slope of 0.8615 ± 0.02436 and R^2^ = 0.8742 (Fig. 4J**)** and this is also improved compared to the IHC analysis that resulted in an R^2^ = 0.59.

### Changes in nuclear density in progressing samples

**Figure 5:**
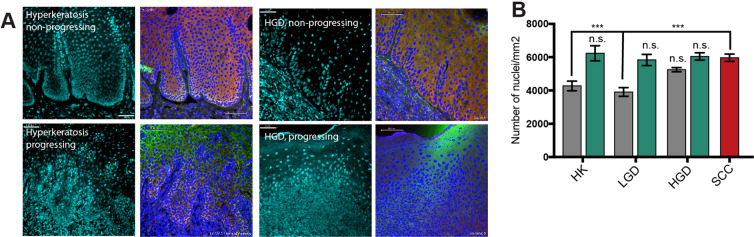
(**A)** Representative FIHC images of control (non-progressing) and progressing hyperkeratosis and HGD showing DAPI channel on the left and merged PD-1(green)/PD-L1(red) on the right. The blue mask represents an automated cell identification protocol by Definiens Tissue Studio 4.0 based in DAPI staining. **(B)** Quantification of cell density per field, ANOVA and Tukey’s test, n = 10 fields per sample, ***P < 0.001.

During the analysis of FIHC images we noticed an increase in the cellularity in progressing samples compared to non-progressing cases. In order to test this, we used an automated tissue phenomics software to calculate cell density. We used the DAPI stained samples to calculate the average number of cells per field using an automated protocol from the manufacturer. The results show a significant increase in cellularity in progressing HK and LGD (P < 0.05) compared to control (Fig. 5). There were no significant differences in cellularity between progressing and non-progressing HGD compared to OSCC.

## Discussion

### Technology development: FIHC automated analysis

In this pilot study, our goal was to show that FIHC analysis with standardized image analysis is feasible, results in an improved interobserver agreement, and should be explored as a tool to determine PD-1 and PD-L1 expression in a clinical setting. Reproducible methods for detecting PD-L1 and PD-1 expression are critical to determine which patients may benefit from targeted anti-PD-1 and PD-L1 therapies^12,25^. Previous studies have shown that interobserver variability is higher than the assay variability and new standardized methods to determine expression are needed^26^. Several approaches have been used to overcome these challenges, including multiplexing and automated staining analysis^28^, chemiluminescent magnetic immunoassays^29^ and flow cytometry-based analyses^30^. Each of these methods have challenges to clinical implementation, including technical complexity and elevated costs. Many factors support FIHC and our proposed analysis over current IHC-based quantification. First, our proposed protocol is based on standard laboratory processing procedures and require no additional laboratory equipment. Also, our analysis only requires the selection of the area of interest by the operator, and the protocol can calculate positive area and MFI, decreasing the risk of bias and improving reproducibility. The linear relationship between expression and signal intensity provided by FIHC can significantly reduce the variability of the results caused by peroxide reaction and is an overall preferable method for objective quantification. After optimization of the protocol, our analysis could be completed within 48 hours after the specimen was embedded, which is similar to processing times of IHC in pathology laboratories. The analysis protocol can be adapted to be used in any image analysis software since detection is based on the standard deviation of the pixel intensity. The only major disadvantage is the need of a confocal microscope, which is not usually available in all hospitals.

McLaughlin *et al*.^31^ has previously compared IHC-based quantification and quantitative immunofluorescence (QIF) methods to determine PD-L1 expression in 49 Non-small Cell Lung Cancer (NSCLC) patients. The authors report a heterogeneous expression, similar to our own observations and up to 25% discordance between PD-L1 antibodies. Differently from NSCLC there are no specific thresholds to determine positivity in OSCC, therefore we could not specify concordance based on thresholds. Further studies are needed to define clinically relevant thresholds in OSCC. Our results also show significant differences in PD-1 IHC and FIHC staining intensity and this may be due to differences in antibody clones that work of IHC and may not work as well for FIHC. Further comparative studies are needed to better understand the differences between available clones.

### PD-1 and PD-L1 in oral cancer progression

Cancer immunosurveillance is an essential protective response that prevents the development of malignant tumors through the early elimination of transformed cells^32^. Early elimination can also contribute to the selection of tumor cells that can evade antitumor responses based on the concept of immunoediting^33,34^. The immune checkpoint system is one of the mechanisms through which tumors can escape antitumor responses evading eradication by the host immune system by attenuating T-cell mediated responses^35,36^. Immunomodulatory monoclonal antibodies, which target the PD-1/PD-L1 pathway, have shown promising results in clinical trials of several cancers^37,38^. However, most of our understanding of PD-1/PD-L1 role in cancer is based on models in which tumors have already escaped immunosurveillance, and very few studies have investigated their roles in pre-cancerous lesions^24,39^. In this context, oral premalignant lesions represent an excellent model for understanding the expression of PD-1/PD-L1 and how this immune checkpoint pathway is involved in malignant transformation for the following two reasons: 1-OED can precede malignant transformation but already shows various genetic abnormalities, including mutations in the P53 gene and differential expression of several genes involved in the regulation of immune responses^40^. 2. OED is commonly associated with an increase in the inflammatory infiltrate, particularly TCD4 cells and neutrophils, which can be seen in direct contact with the epithelial cells^10^. These interactions are occurring primarily at the basal epithelial layer, but invasion is still not present.

A recent study reported an increase in the number of PD-L1 positive cells in oral dysplastic lesions and OSCCs^41^; however, it is well known that not all dysplastic lesions will transform to SCC^42–46^. The significant increase in PD-L1 expression in basal epithelial cells and inflammatory cells in lesions that progress to cancer reported here suggests that the activation of mechanisms that suppress the elimination of transformed cells precede cellular invasion which is the hallmark of cancer. Therefore, our results highlight the importance of the immune responses in early transformation not only mechanistically but as a potential tool to predict and monitor malignant transformation. Considering the morbidity and mortality of oral cancer, this can create a new approach to immunotherapies that will focus on prevention rather than an adjuvant to treatment.

One of the challenges in interpreting PD-1/PD-L1 expression in oral premalignant lesions and OSCC in the constant presence of dense inflammatory infiltrates causing an increase in cell density. As we have shown before, there is an increase in inflammatory cells in HGD and OSCC, particularly neutrophils and TCD4 cells that can promote invasion of cancer cells *in vitro*^10^. Here we used automated analysis to calculate cell density to show an increase in cell density in progressing HK and LGD lesions, while HGD shows similar cell density to OSCC. We interpret these changes as a marked increase in OPMD-associated and tumor-associated inflammatory cells. The increase in inflammatory cells may be a confounding factor to explain the increased PD-L1 expression in these samples, and further studies are needed to determine the exact ratios of PD-1 and PD-L1 inflammatory cells in these lesions.

## Limitations

There are several critical steps to implement a new clinical protocol for biomarker quantification, including standardization of the assay, definition of antibody clones, quantification and clinical correlation. One major limitation is the sample size. Our small sample size reflects the challenges of finding adequate retrospective material for OSCC cases that progress to cancer with enough follow up time. The low number of cases prevents an in-depth analysis of PD-1/PD-L1 expression and clinical correlation, outcomes and potential use as a prognostic marker. This is one of the first studies to assess PD-1/PD-L1 expression in OSCC and premalignant oral lesions, therefore clinical correlation and cut-off values to determine treatment are not available. Future studies are needed to determine the clinical relevance of PD-1/PD-L1 expression in OSCC.

## Conclusion

We report a novel quantifiable, semi-automated FIHC-based method for quantifying the expression of PD-1 and PD-L1 using FFPE samples. Using this method, we show that the PD-1/PD-L1 may be activated early in premalignant lesions, sometimes years before malignant transformation. Within the limitations of a small cohort and retrospective analysis, the results of our study could be used to develop new clinical tools to improve the quantification of PD-1/PD-L1 and identify lesions with a higher risk of progressing to cancer.
